# Insurance, Assurance and Insolence

**Published:** 1898-06

**Authors:** J. L. Jones

**Affiliations:** Gatesville, Texas


### Correspondence.

### "Insurance," "Assurance" and "Insolence."

Perhaps one of the most unique exhibitions of cool insolence, pertaining to "things medical," is that of life insurance companies trying to regulate the compensation of medical practitioners, an instance of which follows: You are approached by a young gentleman wearing a sweet smile, hair carefully parted in the middle, of undue cheek and unmitigated gall. This person informs you that he represents the ——— Insurance Company, who make free medical attendance a feature of their policy, all members being assured of medical services free of charge to themselves. He further proceeds to inform you, without so much as blushing, that his company will pay you two dollars *per capita*, per annum, for members' medical attendance in your vicinity. Should you be willing, he will inform you further that this amount is to be paid quarterly. In addition, one dollar is paid for each member's entrance examination.

Now, if an agent is fairly successful in a town of, say 3000 people, he may secure twenty-five members, which would mean fifty dollars per annum for the company's physician; and for this insignificant sum the physician must place himself at the beck and call of twenty-five persons, some of whom, in all probability, the previous year paid him more than this for individual services, and enter into a thing of this kind merely as a matter of speculation. The particular company I have in view has an entrance examination which, if thoroughly complied with, would occupy hours—urine analysis and microscopical examination of casts—and all to be done for less than the price demanded for a prescription.

When such a state of affairs is reached, is it not time for the profession to call a halt?—refuse to deal with any insurance agent excepting under conditions dictated by the physician himself; refuse to dispense his expensively attained information and scientific knowledge but at his own particular pleasure and price, and otherwise treat him as he would a private individual?

There are perhaps some who will say, "Why, no agent making a proposition of this kind could secure an examiner at such a price;" but it is only too true that they can, and do, in nearly every instance. Surely the time has come when the medical fraternity should say to a company: "We are perfectly willing to make your examinations, but our price is so and so," and scorn to make a contract with a company to do the practice of an individual for a whole year for less than he ordinarily charges for a single visit.

Who would have dared to approach our dignified M. D. of fifty years ago with a similar proposition? The professional man of that day and time would not even have considered such an offer; he guarded his professional privileges jealously, would have scoffed at such insolence as detailed above; neither would he brook interference. And pray why should we do so now? Our information is just as valuable as his was then, and in many respects more practical. Let us begin to remedy this evil by passing suitable resolutions in our associations, and work for a return of the old state of affairs, when examinations, etc., were on a more satisfactory basis of compensation; for if we suffer affairs to take the course they are doing now, in a short time we shall be working for nothing.

J. L. JONES, M. D.

Gatesville, Texas, March 22, 1898.

